# Scalable privacy-preserving data sharing methodology for genome-wide association studies: an application to iDASH healthcare privacy protection challenge

**DOI:** 10.1186/1472-6947-14-S1-S3

**Published:** 2014-12-08

**Authors:** Fei Yu, Zhanglong Ji

**Affiliations:** 1 Department of Statistics, Carnegie Mellon University, 5000 Forbes Ave, PA 15213, Pittsburgh, PA, USA; 2 Department of Computer Science and Engineering, University of California, San Diego, CA 92092, La Jolla, CA, USA

**Keywords:** *χ*^2 ^statistic, Contingency table, Differential privacy, Genome-wide association study GWAS, Data sharing, Single-nucleotide polymorphism

## Abstract

In response to the growing interest in genome-wide association study (GWAS) data privacy, the Integrating Data for Analysis, Anonymization and SHaring (iDASH) center organized the *iDASH Healthcare Privacy Protection Challenge*, with the aim of investigating the effectiveness of applying privacy-preserving methodologies to human genetic data. This paper is based on a submission to the iDASH Healthcare Privacy Protection Challenge. We apply privacy-preserving methods that are adapted from Uhler et al. 2013 and Yu et al. 2014 to the challenge's data and analyze the data utility after the data are perturbed by the privacy-preserving methods. Major contributions of this paper include new interpretation of the *χ*^2 ^statistic in a GWAS setting and new results about the Hamming distance score, a key component for one of the privacy-preserving methods.

## Introduction

Rapid developments in whole-genome sequencing technologies in recent years have made the collection of high quality genetic data faster and more economically feasible. Many types of genetic research can benefit from having a large amount of genetic data. For example, in genome-wide association studies (GWAS), which are a type of genetic research that examine a large number of single-nucleotide polymorphisms (SNPs) to identify genetic factors associated with a phenotype, which is typically a common disease, increasing the number of DNA samples available for analysis allows researchers to make more accurate statistical inference and improve the overall quality of the analysis.

Encouraging data sharing among researchers is the first step towards taking advantage of the benefits brought about by the rapid growth in genetic data collection. However, being able to share genetic data without compromising the study participants' privacy remains one of the biggest challenges in genetic research. While it is clear that individual level genetic data deserve a high level of protection, for many years it was widely considered safe to release to the public aggregate genetic data pooled from thousands of individuals without compromising genetic study participants' privacy. However, Homer et al. [1] in 2008 demonstrated that one can use publicly available aggregate genetic data, such as SNP data from the International HapMap Project http://hapmap.ncbi.nlm.nih.gov/, to infer whether an individual has participated in a study. Cautious about the potential breach of genetic study participants' privacy, the National Institute of Health (NIH) quickly responded to the Homer et al. [1] attack by mandating an elaborate approval process that every researcher has to go through in order to gain access to aggregate genetic data. This NIH policy remains in effect today.

Homer et al. [1]'s attack and NIH's subsequent reaction spurred research interest in privacy-preserving methodologies for GWAS data. A recent concept of *differential privacy *(e.g. [2]), introduced by the cryptographic community, has shown great promise as a basis for privacy-preserving methodologies, as it provides a rigorous definition of privacy with meaningful privacy guarantees in the presence of arbitrary external information. We have seen privacy-preserving methods based on differential privacy applied to real human GWAS data in recent studies (e.g., [3-5]).

The iDASH Healthcare Privacy Protection Challenge, organized by *Integrating Data for Analysis, Anonymization and SHaring *(iDASH), aims to investigate the effectiveness of applying privacy-preserving methodologies to human genetic data [6]. This paper is based on a submission to the iDASH Healthcare Privacy Protection Challenge using privacy-preserving methods adapted from [3] and [5].

A major contribution of this paper is a new interpretation of the *χ*^2 ^statistic in a GWAS setting and new results about the Hamming distance score, which plays an important role in the differentially private mechanisms proposed by [4] and [5]. In particular, we present a graphical interpretation of the allelic test *χ*^2 ^statistic that will help us conceptualize the Hamming distance score. We also device an efficient algorithm for finding the Hamming distance score and prove that the *sensitivity *of the score function is 1; we hence address concerns raised in [5] about speed and sensitivity of alternative methods for finding the Hamming distance score.

We start by introducing background information on the iDASH Healthcare Privacy Protection Challenge. We briefly describe the characteristics of the data and define the allelic test *χ*^2 ^statistic, which is used for evaluating the performance of submissions in the challenge. Then we summarize differentially private mechanisms applied to the challenge's data, which include a mechanism based on the Laplace mechanism and *χ*^2 ^statistic, a mechanism based on the exponential mechanism and *χ*^2 ^statistic, and a mechanism based on the exponential mechanism and Hamming distance score. We present a graphical interpretation of the allelic test *χ*^2 ^statistic and an efficient algorithm for finding the Hamming distance score. We prove that our algorithm finds the shortest Hamming distance and therefore the Hamming distance score has sensitivity 1. We incorporate our improvements into the differentially private mechanisms and apply them to the challenge's data. We compare the performance of the mechanisms using risk-utility plots.

## Background information on iDASH challenge

The challenge has two tasks, both of which are concerned with the dissemination of aggregate GWAS data: (1) limiting the re-identification risks when releasing all aggregate data in a GWAS dataset, and (2) being compliant with differential privacy (Definition 2) when releasing the most significant SNPs. This paper focuses on the second task of releasing the most significant SNPs differentially privately.

The data used for the second task consist of 201 participants from the Personal Genome Project (http://www.personalgenomes.org/) and 174 participants from HapMap. Individuals from PGP are treated as cases and those from HapMap are treated as controls in the challenge. 106,129 SNPs are typed in all participants. [6] has more details on how the data are processed.

A subset containing 5,000 SNPs is selected by organizers of the challenge to form a representative sample of the entire set of SNPs. This paper uses the subset of SNPs to evaluate the performance of the privacy-preserving methods, as is recommended by organizers of the challenge.

In GWAS with *R *cases and *S *controls, we usually summarize the data for a single SNP using a 2 × 3 genotype contingency table shown in Table 1 or a 2 × 2 allelic contingency table shown in Table 2. In this challenge, we are given genotypes of individuals in the case group and allele frequencies of individuals in the control group. Therefore, the row pertaining to the case group in the allelic table can be easily derived from the genotypes of the case group. Furthermore, we will assume that the Hardy-Weinberg equilibrium holds so that we can derive the row pertaining to the control group in the genotype table from the allele frequencies of the control group.

**Table 1:** Genotype table

	# of minor alleles			Total
		
	0	1	2	
Case	*r*_0_	*r*_1_	*r*_2_	R
Control	*s*_0_	*s*_1_	*s*_2_	S
Total	*n*_0_	*n*_1_	*n*_2_	N

**Table 2:** Allelic table

	Allele type		Total
		
	Minor	Major	
Case	*r*_1 _+ 2*r*_2_	2*r*_0 _+ *r*_1_	2R
Control	*s*_1 _+ 2*s*_2_	2*s*_0 _+ *s*_1_	2S
Total	*n*_1 _+ 2*n*_2_	2*n*_0 _+ *n*_1_	2N

In this challenge, the statistical significance of a SNP's association with the phenotype is assessed by the the allelic test statistic (Definition 1). For the rest of the paper, we will simply refer to the allelic test statistic as *χ*^2 ^statistic. Assuming that the control group's data are public, we will use the differentially private mechanisms discussed in the next section to release the top *K *SNPs while preserving the privacy of the case group.

**Definition 1 ***The allelic test is also known as the Cochran-Armitage trend test for the additive model. The allelic test statistic based on a genotype contingency table (Table *1*) is equivalent to the χ*^2^*-statistic based on the corresponding allelic contingency table (Table *2*). The allelic test statistic can be written as*

$$Y_{A}=\frac{2N{\left[\left(2r_{0}+r_{1}\right)S-\left(2s_{0}+s_{1}\right)R\right]}^{2}}{RS\left(2n_{0}+n_{1}\right)\left(n_{1}+2n_{2}\right)}$$

## Differential privacy: definitions and methods

The concept of differential privacy, recently introduced by the cryptographic community (e.g., [2]), provides a notion of privacy guarantees that protect GWAS databases against arbitrary external information.

**Definition 2 **(differential privacy) *Let $D=\left\{\left(X_{1},\ldots ,X_{n}\right):X_{i}\sim P\right\}$ denote the set of all databases consisting of n individuals sampled independently from the same population  P. For $D,D^{\prime }\in D$, write D ~ D' if D and D' differ in one individual. A randomized mechanism  K is ∈*-differentially private *if, for all D ~ D' and for any measurable set S⊂ℝ,*

$$\frac{Pr\left(K\left(D\right)\in S\right)}{Pr\left(K\left(D^{\prime }\right)\in S\right)}\leq e^{\varepsilon }.$$

Two methods are often used as building blocks for constructing more complex differentially private algorithms. One of the methods, due to [2], is called the *Laplace mechanism *(Definition 4), and the other method, due to [7], is called the *exponential mechanism *(Definition 5). Both methods require knowledge of the *sensitivity *of the score function, where sensitivity is defined as the smallest upper bound of how much the function can vary when one record in the input database changes (see Definition 3).

**Definition 3 ***The *sensitivity *of a function $f:D\times {\mathbb{R}}^{d}\to \mathbb{R}$ is the smallest number S*(*f*) *such that*

$$\underset{x\in {\mathbb{R}}^{d}}{\text{sup}}||f\left(D,x\right)-f\left(D^{\prime },x\right)||\leq S\left(f\right),$$

*for all databases *$D,D^{\prime }\in D$*such that D ~ D'*.

**Definition 4 **(Laplace mechanism) *Releasing f *(*D*)+*b, where b ~ Laplace $\left(0,\frac{S\left(f\right)}{\varepsilon }\right)$, satisfies the definition of ∈-differential privacy*.

**Definition 5 **(exponential mechanism) *Let $q:D\times {\mathbb{R}}^{d}\to \mathbb{R}$ be a function that outputs the score of an event or a value given a database. Define the random variable *$\varepsilon_{q}^{\varepsilon }$

$$\text{Pr}\left(\varepsilon_{q}^{\varepsilon }\left(D\right)=x\right)=\frac{\text{exp}\left(\frac{\varepsilon q\left(D,x\right)}{2S\left(q\right)}\right)}{\int_{{\mathbb{R}}^{d}}\text{exp}\left(\frac{\varepsilon q\left(D,s\right)}{2S\left(q\right)}\right)\text{d}s}.$$

*Then releasing $\varepsilon_{q}^{\varepsilon }$ satisfies the definition of ∈-differential privacy*.

## Methods for releasing the *K *most relevant SNPs

**Algorithm 1 **The *E*-differentially private mechanism for releasing the *K *most relevant SNPs using the *Laplace mechanism *[3,5,8].

**Input: **The score of all *M *candidate SNPs, the number of SNPs, *K*, that we want to release, the sensitivity, *s*, of the score function, and the privacy budget *E*.

**Output: ***K *SNPs.

1: Add independent Laplace noise with mean zero and scale $\frac{2K\;s}{\varepsilon }$ to each of the *M *SNPs scores.

2: Choose the top *K *SNPs based on the perturbed scores.

**Algorithm 2 **The *∈*-differentially private mechanism for releasing the *K *most relevant SNPs using the *exponential mechanism *[4,5].

**Input: **The scores (e.g. *χ*^2 ^statistic or Hamming distance) of all *M *candidate SNPs, the number of SNPs, *K*, that we want to release, the sensitivity, *s*, of the score function, and the privacy budget *∈*.

**Output: ***K *SNPs.

1: Initialize ${\left\{q_{i}\right\}}_{i=1}^{M}$ score of SNP*_i_*.

2: Set $w_{i}=\text{exp}\left(\frac{\varepsilon q_{i}}{2Ks}\right)$. Define $\text{Pr}\left(T\left(D\right)=i\right)=w_{i}/\overset{M}{\underset{j=1}{\sum }}w_{j}.$

3: Sample j~T(D). Record SNP*_j _*. Set *q_j _*= *−∞*.

4: Repeat Step 2 and 3 until *K *SNPs have been recorded.

Algorithm 1 and Algorithm 2 extend the Laplace mechanism and the exponential mechanism, respectively, to release more than a single SNP differentially privately. In this paper, we consider three mechanisms for releasing the top *K *SNPs: a mechanism that is based on Algorithm 1 and uses *χ*^2 ^statistic as score function, a mechanism that is based on Algorithm 2 and uses *χ*^2 ^statistic as score function, and a mechanism that is based on Algorithm 2 and uses the Hamming distance score ([4]) as score function. In loose terms, the Hamming distance score is the smallest number of changes made to a genotype table until the significance of the table changes, where a change, counted as 1-Hamming distance in the space of genotype tables, is defined as changing the genotype of one individual and significance refers to whether the *p*-value of the *χ*^2 ^statistic of the table is smaller than a pre-specified threshold value or not. See [5] for more details on the three mechanisms and applications of them to a real human GWAS dataset.

For mechanisms that use the *χ*^2 ^statistic as score, we need to know the sensitivity of the *χ*^2 ^statistic. An upper bound for the sensitivity is shown in [5], but [5] requires that the margins of the genotype contingency tables to be positive. Indeed, such requirement can be satisfied in the challenge's setting when we assume that Hardy-Weinberg equilibrium holds: because a typical GWAS dataset consists of only common SNPs, whose minor allele frequencies are greater than 1%, the control group's three genotypes derived from the allele frequency at each SNPs will be nonnegative, which ensures that the derived genotype tables have positive margins.

For the mechanism that uses the Hamming distance score as score, we already know that, by construction, the sensitivity of the score function is 1 if the Hamming distance is the shortest Hamming distance [4]. However, as is pointed out in [4] and [5], it is a computationally onerous task to actually calculate the shortest Hamming distance, which, in the most naïve setting, involves examining all possible sequential changes made to the original genotype table that alter the significance status of the table. To make the calculations more computationally feasible, [4] and [5] use approximations of the shortest Hamming distance in their implementations of the mechanism, noting the caveat that the sensitivity of the approximated Hamming distance score may no longer be 1.

In the next section, we propose a new method of finding the Hamming distance score that is much more computationally efficient than those in [4] and [5]. We also prove that our method indeed produces the shortest Hamming distance, and therefore the sensitivity of the resulting Hamming distance score function is 1.

## Finding the Hamming distance score

**Figure 1:**
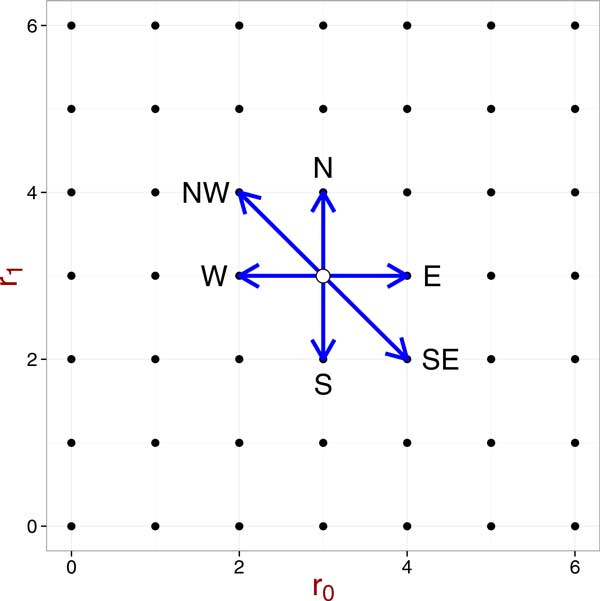
**Legal moves in the space of genotype tables with fixed *R, S, s*_0_*, s*_1_, and *s*_2_**.

Let's refer to the case group's data and the control group's data collectively as a database and call the data for an individual a record. We can think of the number of cases, *R*, and the number of controls, *S *as fixed. Recall that we assume the control group's data are known to the public. Therefore, for a given genotype table, we assume that *s*_0_, *s*_1_, and *s*_2 _are fixed. Then the *χ*^2 ^statistic can be written as a function of *r*_0 _and *r*_1_. How the value of the *χ*^2 ^statistic changes when we change one record in the database is illustrated in Figure 1. In Figure 1, each dot represent a value of the *χ*^2 ^statistic given *r*_0 _and *r*_1_. When we change one record in the case group, there are 6 possible changes to the genotype table: (*r*_0 _*→ r*_0 _+ 1*, r*_1 _*→ r*_1_), (*r*_0 _*→ r*_0 _+ 1*, r*_1 _*→ r*_1 _*− *1), (*r*_0 _*→ r*_0_*, r*_1 _*→ r*_1 _*− *1), (*r*_0 _*→ r*_0 _*− *1*, r*_1 _*→ r*_1_), (*r*_0 _*→ r*_0 _*− *1*, r*_1 _*→ r*_1 _+ 1), and (*r*_0 _*→ r*_0_*, r*_1 _*→ r*_1 _+ 1); that is, *r*_0 _and *r*_1 _cannot increase or decrease by 1 simultaneously. The possible changes are shown as arrows in Figure 1. A change in the genotype table results in a change in the allelic table, and we get a new value for the *χ*^2 ^statistic based on the new allelic table.

Let p^* ^denote a pre-specified threshold *p*-value and let *c *denote the *χ*^2 ^statistic corresponding to *p^∗^*, the *p*-value of the *χ*^2 ^distribution with 1 degree of freedom. Then for a given SNP in the pool of candidate SNPs, the genotype table of which we denote by *D*, the shortest Hamming distance is the smallest number of sequential changes made to *D *such that the resulting genotype table, *D'*, satisfies *Y_A_*(*D'*) *≥ c *if *Y_A_*(*D*) *< c *and *Y_A_*(*D'*) *< c *if *Y_A_*(*D*) *≥ c*; that is, if we call *c *the significance threshold, then the goal is to make changes to the "insignificant" ("significant") table *D *so that the *χ*^2 ^statistic of *D' *goes above (below) the significance threshold *c*, and *D' *becomes a "significant" ("insignificant") table. The Hamming distance score is defined as *h *= (shortest Hamming distance) *− *1 if *Y_A_*(*D*) *≥ c *and *h *= *−*(shortest Hamming distance) if *Y_A_*(*D*) *< c*.

Let's consider the space of genotype tables, $B_{D}$, defined by a genotype table *D*: for all $D^{\prime }\in B_{D}$, *D' *shares the same values of *s*_0_, *s*_1_, *s*_2_, *R, S*, and *N *with *D*, but *D' *does not necessarily have the same values of *r*_0_, *r*_1_, and *r*_2 _as *D*. Let *n*_10 _= 2*s*_0 _+ *s*_1 _denote the number of major alleles in the control group, and let *x *= 2*r*_0 _+ *r*_1 _denote the number of major alleles in the control group, then we can write the *χ*^2 ^statistic of a genotype table $D^{\prime }\in B_{D}$ as a function of *x*:

$$\begin{array}{ccc} Y_{A}\left(D;B_{D}\right) & = & Y\left(r_{0},r_{1}:D\right)=Y_{A}\left(x;D\right)\\ & = & \frac{2N{\left(xS-n_{10}R\right)}^{2}}{RS\left(x+n_{10}\right)\left(2N-x-n_{10}\right)}, \end{array}$$

where *r*_0_, *r*_1 _and *x *are derived from *D'*, and *n*_10_, *R, S *and *N *are the same for *D *and *D^l^*. For notational convenience, when *r*_0 _and *r*_1 _are also derived from *D*, we will simply write the *χ*^2 ^statistic as *Y_A_*(*D*).

**Lemma 1 ***Y_A _is an increasing function of × when xS − n*_10_*R >*0*, and it is a decreasing function of × when xS − n*_10_*R <*0.

*Proof*. [see Additional file 1].

**Figure 2:**
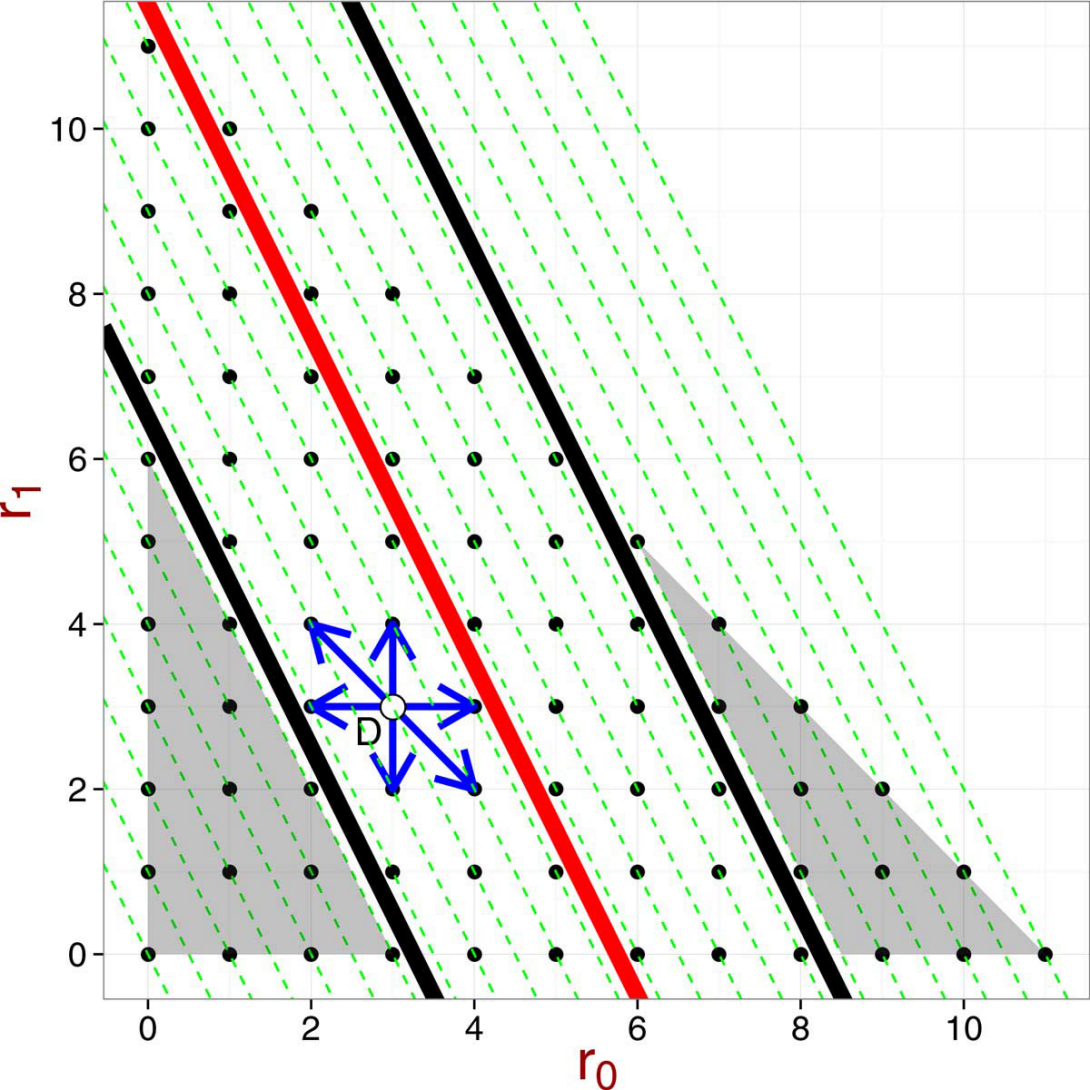
**An example of a genotype table, *D*, in the space of genotype tables with fixed *R, S, s*_0_, *s*_1_, and *s*_2_**. Each dot represent a genotype table. Each dashed line has *slope *= fl2, representing the lines *x *= 2*r*_0 _+ *r*_1_. The red line is *x *= (2*s*_0 _+ *s*_1_)*R/S *= 2*r*_0 _+ *r*_1_, and the two black lines correspond to values of (2*r*_0 _+ *r*_1_) such that *Y_A_*(*r*_0_*, r*_1_; $B_{D}$) = *c*, where *c *is a pre-specified significance threshold value.

To understand the implication of Lemma 1, let's consider Figure 2. In Figure 2, each dashed line has *slope *= *−*2, representing a value of *x*, which is defined as *x *= 2*r*_0 _+ *r*_1_. Because we can consider each dot in Figure 2 to be a unique genotype table in the space of genotype tables with fixed control data and a fixed number of cases, those tables that lie on the same dashed line will have the same value of *χ*^2 ^statistic. Furthermore, because 0 *≤ r*_0 _+ *r*_1 _*≤ R, r*_0 _*≥ *0 and *r*_1 _*≥ *0, the space of genotype tables, represented as dots, fall within a triangle in Figure 2.

For the moment, let's treat *r*_0 _and *r*_1 _as continuous values. In Figure 2, the red solid line represents the line 2*r*_0 _+ *r*_1 _= *x *= *n*_10_*R/S *and the two solid black lines represent lines 2*r*_0 _+ *r*_1 _= *x *such that $Y_{A}\left(D;B_{D}\right)=c$. There are two black lines because by Lemma 1 *Y_A_*(*x*) is an increasing function when *x > n*_10_*R/S *and it is a decreasing function when *x < n*_10_*R/S*; that is, there could be two values of *x*, say *x*_1 _and *x*_2_, such that $Y_{A}\left(x_{1};B_{D}\right)=Y_{A}\left(x_{2};B_{D}\right)$ and *x*_1 _*< n*_10_*R/S < x*_2_. Because it is possible that

$$\begin{array}{c} \;\;\;\;\underset{x}{\text{max}}Y_{A}\left(x;D\right)\\ \leq \text{max}\left\{Y_{A}\left(0,B_{D}\right),Y_{A}\left(2R,B_{D}\right)\right\}\\ =\text{max}\left\{\frac{2NRn_{10}}{S\left(2N-n_{10}\right)},\frac{2NR\left(2S-n_{10}\right)}{S\left(2R+n_{10}\right)}\right\}<c, \end{array}$$

there could be genotype tables for which only one black line exists or no black line exists at all; in such cases, we will use the lines 0 = 2*r*_0 _+ *r*_1 _or 2*R *= 2*r*_0 _+ *r*_1 _wherever appropriate.

In Figure 2, the genotype table *D *is insignificant and its *χ*^2 ^statistic is below the threhold value. By Lemma 1, we know that the *χ*^2 ^statistics of genotype tables, as represented by the dots on Figure 2, are greater than *c *when they are in the shaded area, outside of the area between the two black lines and they are smaller than *Y_A_*(*D^∗^*) when they are inside the area between the two black lines. Therefore, finding the Hamming distance score for *D *is to find the shortest Hamming distance from the genotype table *D *to genotype tables in the shaded areas.

For genotype tables that are significant, they will fall into the shaded areas in Figure 2. Then finding the Hamming distance score for a significant genotype table is to find the shortest Hamming distance from the genotype table in one of the shaded areas to genotype tables in the non-shaded area.

**Proposition 2 ***Given a significance threshold value c and an insignificant genotype table D (i.e., Y_A_*(*D*) *< c), if there exists $D^{\prime }\in B_{D}$ such that $Y_{A}\left(D^{\prime };B_{D}\right)\geq c$, then the shortest Hamming distance is *min{*H*_1_*, H*_2_}*, where H*_1 _*and H*_2 _*are defined as follows:*

*(i) H*_1 _*is the number of changes made to D in the following manner: (1) keep decreasing r*_0 _*until the new genotype table, D', becomes significant (i.e.,$Y_{A}\left(D^{\prime };D\right)>c$); (2) when r*_0 _*is minimized but the new table is still insignificant, keep decreasing r*_1 _*until the new table becomes significant*.

*(ii) H*_2 _*is the number of changes made to D in the following manner: (1) keep increasing r*_0 _*until the new genotype table becomes significant; (2) if r*_0 _*can no longer be increased without decreasing r*_1 _*and the new table is still insignificant, increase r*_0 _*and decrease r*_1 _*in each change until the new table becomes significant*.

*If for all $D^{\prime }\in B_{D}$, $Y_{A}\left(D^{\prime };B_{D}\right)<c$, then we define the shortest Hamming distance as *min$\left\{H_{1}^{\prime },H_{2}^{\prime }\right\}$*, where $H_{1}^{\prime }$ and $H_{2}^{\prime }$ are defined as follows:*

*(i) When r*_0 _*and r*_1 _*are both minimized but the new table is still insignificant, set $H_{1}^{\prime }$to *1 + *d*_1_*, where d*_1 _*is smallest the number of changes needed to minimize r*_0 _*and r*_1_.

*(ii) When r*_0 _*and r*_1 _*are both maximized but the new table is still insignificant, set $H_{2}^{\prime }$to *1 + *d*_2_*, where d*_2 _*is smallest the number of changes needed to maximize r*_0 _*and r*_1_.

*Proof*. [see Additional file 1].

**Proposition 3 ***Given a significance threshold value c and a significant genotype table D (that is, Y_A_*(*D*) *≥ c), the shortest Hamming distance is *min{*H*_1_*, H*_2_}*, where H*_1 _*and H*_2 _*are defined as follows:*

*(i) If *2*r*_0 _+ *r*_1 _*>*(2*s*_0 _+ *s*_1_)*R/S, set H*_1 _= *∞; otherwise, H*_1 _*is the number of changes made to D in the following manner: keep decreasing r*_0 _*until the new genotype table, D', becomes insignificant (i.e., Y_A_*(*D', D*) *< c)*.

*(ii) If *2*r*_0 _+ *r*_1 _*<*(2*s*_0 _+ *s*_1_)*R/S, set H*_2 _= *∞; otherwise, H*_2 _*is the number of changes made to D in the following manner: keep decreasing r*_0 _*until the new genotype table becomes insignificant*.

*Proof *The proof is similar to that of Proposition 2.

**Definition 6 **(The Hamming distance score) *Given a threshold χ*^2 ^*statistic value c and a genotye table D, the Hamming distance score of D is*

$$h\left\{\begin{array}{cc} -d^{-}, & if\;Y_{A}\left(D\right)<c,\\ d^{+}-1 & if\;Y_{A}\left(D\right)\geq c, \end{array}\right)$$

*where d^− ^is found using Proposition 2 and d*^+ ^*is found using Proposition 3*.

**Corollary 4 ***The sensitivity of the Hamming distance score as defined in Definition 6 is 1*.

## Application to the challenge's data

In this section we apply all three differentially private mechanisms to the challenge's data and evaluate the performace of the mechanims by examining the data utility at several levels of privacy risk. Data utility is defined as follows: let $S_{0}$ denote the set of top *K *SNPs ranked according to their true *χ*^2 ^statistics and let  S be the set of top *K *SNPs chosen after perturbation (either by Algorithm 1 or Algorithm 2). Then the data utility as a function of the privacy budget, *∈*, is

$$u\left(\varepsilon \right)=\frac{\left|S_{0}\cap S\right|}{\left|S_{0}\right|}.$$

**Figure 3:**
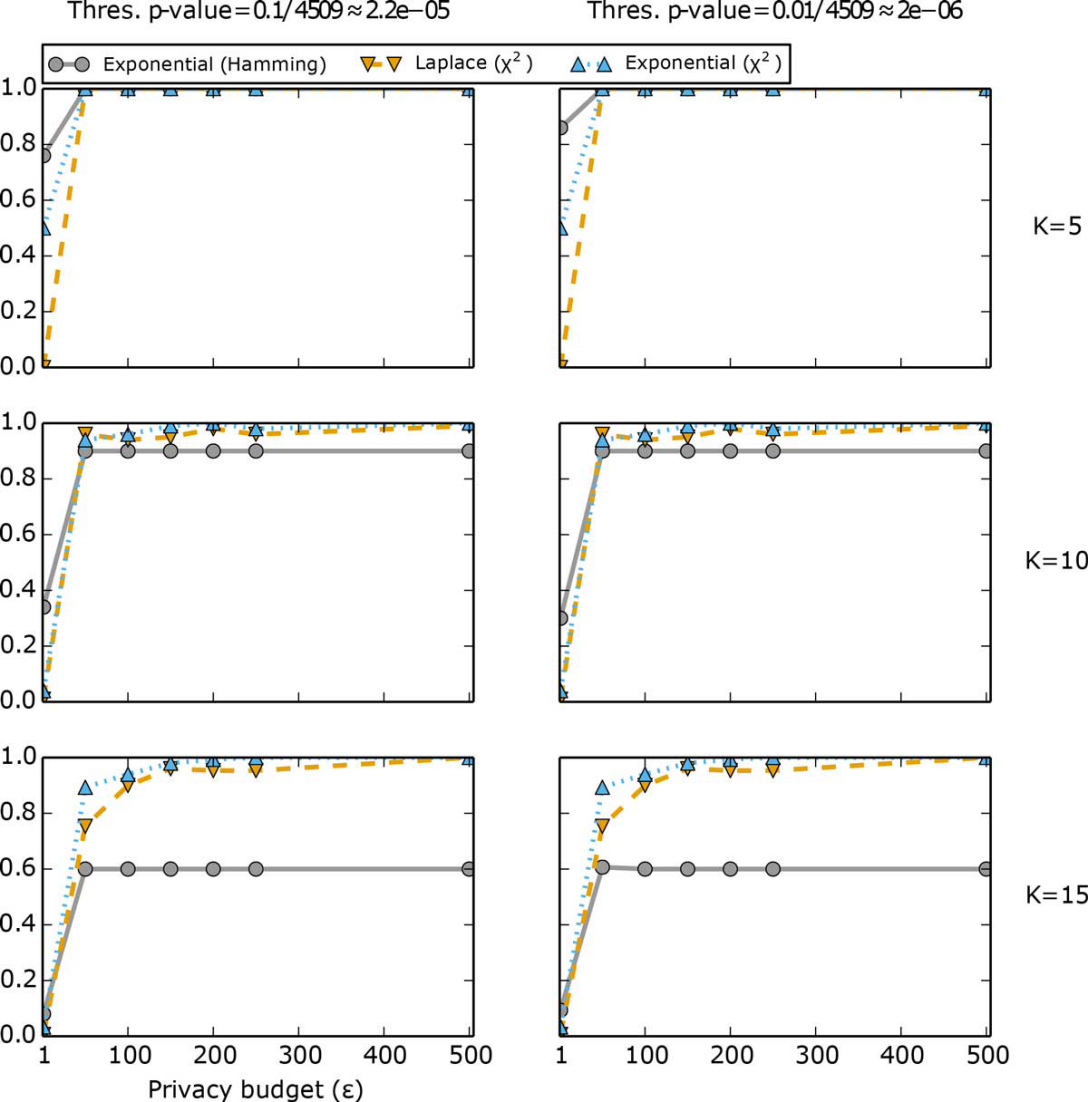
**Risk-utility plots**. Performance comparison of Algorithm 1 and Algorithm 2 with *χ*^2 ^statistic or Hamming distance score as score function. Each row corresponds to a fixed *K*, the number of top SNPs to release. Each column corresponds to a fixed threshold *p*-value, which is relevant to the mechanism based on Hamming distance score only. The threshold *p*-values 0.1 and 0.01 divided by the number of SNPs.

In Figure 3 we compare the performace of the mechanisms given different privacy budget *E *and different number of top SNPs to release, *K*. For the mechanism based on the Hamming distance score, we also consider different significance threshold values. We can see that the mechanism based on Algorithm 2 (a generalization of the exponential mechanism) and the Hamming distance score outperforms the other mechanisms when *∈ *is small (*∈ *= 1); on the other hand, unlike the other mechanisms, the data utilities of which continue to increase as *E *increases, the data utility of the mechanism based on Algorithm 2 and the Hamming distance score may plateau before it reaches 1 even if we keep increasing *E*. This phenomenon is also observed in the analysis of a different GWAS dataset in [5]. The abnormality of the mechanism based on the Hamming distance score is due to the inconsistency in ranking: because the set of top *K *SNPs based on the Hamming distance score is not always the same as the set of top *K *SNPs based on the *χ*^2 ^statistic, which is used to evaluate utility, therefore, as *E *increases, the amount of noise decreases, and the set of *K *SNPs resulting from the mechanism based on the Hamming distance score becomes more similar to the set of top *K *SNPs based on the Hammming distance score, which may depart from the set of SNPS based on the *χ*^2 ^statistic. [5] has a more detailed discussion of the characteristics of all three differentially private mechanisms.

It is also worth noting that even though the performance of the mechanism based on the Hamming distance score does not seem to be affected by the choice of threshold *p*-value, the analysis of the mechanism in [5] shows that whether the choice of threshold *p*-value has any effect on data utility also depends on the choice of *K*, the number of top SNPs to release. Therefore, the choice of threshold *p*-value should be justified before we use this mechanism.

## Conclusions

In our submission to the iDASH Healthcare Privacy Protection Challenge, we apply differentially-private methods proposed by [3] and [5] to the challenge's data. Our results show that the performance of the method based on Algorithm 2 and Hamming distance score is superior to that of other methods when the privacy budget, *∈*, is small. But we also point out problems with the Hamming distance score, such as the data utility plateauing at a level lower than other methods.

We devise an efficient algorithms for finding the Hamming distance score and prove that the sensitivity of the score function is 1. This addresses concerns raised in [5] regarding speed and sensitivity of alternative methods for finding the Hamming distance score. The graphical interpretation of the *χ*^2 ^statistic that we present in the paper is instrumental in our discovery of the efficient algorithm for finding the Hamming distance score. We expect that the graphical interpretation can be extrapolated to other settings, such as the Pearson's *χ*^2 ^statistic for 2 × 3 contingency tables and the setting in which data for the controls are not assumed to be public, and help with designing efficient algorithms for fining the Hamming distance score in those settings.

## Competing interests

The authors declare that they have no competing interests.

## Authors' contributions

Fei Yu participated in the competition, performed the statistical analysis, and drafted the manuscript. Fei Yu and Zhanglong Ji both made significant contributions to devising the method for finding the Hamming distance score. Zhanglong Ji also participated in the revision of the manuscript. All authors read and approved the final manuscript.

## Supplementary Material

Additional file 1 Proofs Click here for file
