# Supplementary material for: Scalable privacy-preserving data sharing methodology for genome-wide association studies: an application to iDASH healthcare privacy protection challenge
Source: BMC Med Inform Decis Mak. 2014 Dec 8;14(Suppl 1):S3. doi: 10.1186/1472-6947-14-S1-S3 (PMC4290802; doi:10.1186/1472-6947-14-S1-S3)
Supplement: Additional file 1 — Proofs [file 1472-6947-14-S1-S3-S1.PDF]

Proof of Lemma 1

$$\frac{\partial}{\partial x}Y_A = \frac{2N}{RS}\frac{1}{(x+n_{10})^2(2N-x-n_{10})^2}C(x),$$

where

$$\begin{aligned}
C(x) &= 2S(xS-n_{10}R)(x+n_{10})(2N-x-n_{10}) - (xS-n_{10}R)^2\left[(2N-x-n_{10})-(x+n_{10})\right] \\
&= (xS-n_{10}R)(2N-x-n_{10})\Big[S(x+n_{10})-(xS-n_{10}R)\Big] \\
&\quad + (xS-n_{10}R)(x+n_{10})\Big[S(2N-x-n_{10})+(xS-n_{10}R)\Big] \\
&= (xS-n_{10}R)\Big[(2N-x-n_{10})n_{10}N + (x+n_{10})(2S-n_{10})N\Big].
\end{aligned}$$

Because $2N-x-n_{10} > 0$ and $2S-n_{10} \geq 0$, therefore $\frac{\partial}{\partial x}Y_A > 0$ when $x > n_{10}R/S$, and $\frac{\partial}{\partial x}Y_A < 0$ when $x < n_{10}R/S$. $\square$

Proof of Proposition 2

We will only prove the first part of Proposition 2 and show that when a significant genotype table exists the first part indeed yields the shortest Hamming distance; the second part of Proposition 2 is designed to handle the extreme cases and does not need proof.

Denote the number of changes made to the insignificant genotype table D until it becomes a significant table D' in each possible direction by $v_E, v_{SE}, v_S, v_W, v_{NW}, v_N$, where the subscripts indicate the direction of change described in Figure 1; that is,

$$\begin{aligned}
E &: (r_0 \to r_0+1, \quad r_1 \to r_1) \\
SE &: (r_0 \to r_0+1, \quad r_1 \to r_1-1) \\
S &: (r_0 \to r_0, \quad r_1 \to r_1-1) \\
W &: (r_0 \to r_0-1, \quad r_1 \to r_1) \\
NW &: (r_0 \to r_0-1, \quad r_1 \to r_1+1) \\
N &: (r_0 \to r_0, \quad r_1 \to r_1+1).
\end{aligned}$$

Let x_L and x_R denote the values of the χ^2 statistic represented by the left and the right black lines, respectively, in Figure 2. Let x_0 denote the value of x on which D resides. When we move the table D to the shaded area to the left of the black lines, we will immediately stop moving D when it becomes a table D' that resides on the line $2r_0 + r_1 = \lfloor x_L \rfloor$. Similarly for the shaded area to the right of the black lines, we immediately stop moving D when it becomes a table D'' that resides on the line $2r_0 + r_1 = \lceil x_R \rceil$. Observe that the number of dotted lines $2r_0 + r_1 = x$, representing discreet values of x, between D and the line on which D' reside is $x - \lfloor x_L \rfloor - 1$, and that between D and the line on which D'' reside is $\lceil x_R \rceil - x - 1$. Also observe that moving in the direction of E, SE, S, W, NW, N results in a change of $2r_0 + r_1$ in the direction of $2, 1, -1, -2, -1, 1$, respectively.

Let's first find the shortest Hamming distance from the table D, represented by the point (r_0, r_1), to the shaded area to the left of the black lines. Finding the shortest

Hamming distance is equivalent to solving the following optimization problem:

$$\begin{aligned}
\text{minimize:} \quad & v_E + v_{SE} + v_S + v_W + v_{NW} + v_N \\
\text{subject to:} \quad & -2v_E - v_{SE} + v_S + 2v_W + v_{NW} - v_N \geq (2r_0 + r_1) - \lfloor x_L \rfloor \\
& -2v_E - v_{SE} + v_S + 2(v_W - 1) + v_{NW} - v_N < (2r_0 + r_1) - \lfloor x_L \rfloor \\
& v_W + v_{NW} - v_E - v_{SE} \leq r_0 - r_0^{\min} \\
& v_S + v_{SE} - v_N - v_{NW} \leq r_1 - r_1^{\min} \\
& v_E, v_{SE}, v_S, v_W, v_{NW}, v_N \geq 0,
\end{aligned}$$

where $r_0^{\min}$ and $r_1^{\min}$ are the smallest possible values for r_0 and r_1, respectively. Because of the requirement that the margins of the genotype table to be positive, $r_0^{\min}$ and $r_1^{\min}$ can be greater than 0. The first constraint ensures that D crosses or ends up on the black line on the left. The second constraint ensures that D does not end up too far from the black line; i.e., moving D to the east by 1 step will prevent D from crossing the black line. The third and fourth constraint ensure that D stays inside the grid does not move past the $r_0^{\min} = 0$ and $r_1^{\min} = 0$ lines, respectively. Let's rewrite the optimization problem as the following:

$$\begin{aligned}
\text{minimize:} \quad & v_E + v_{SE} + v_S + v_W + v_{NW} + v_N \\
\text{subject to:} \quad & 2v_E + v_{SE} - v_S - 2v_W - v_{NW} + v_N + (2r_0 + r_1) - \lfloor x_L \rfloor \leq 0 \\
& 2v_E + v_{SE} - v_S - 2(v_W - 1) - v_{NW} + v_N + (2r_0 + r_1) - \lfloor x_L \rfloor - 1 \leq 0 \\
& -v_E - v_{SE} + v_W + v_{NW} - r_0 + r_0^{\min} \leq 0 \\
& v_{SE} + v_S - v_{NW} - v_N - r_1 + r_1^{\min} \leq 0 \\
& -v_E \leq 0 \\
& -v_{SE} \leq 0 \\
& -v_S \leq 0 \\
& -v_W \leq 0 \\
& -v_{NW} \leq 0 \\
& -v_N \leq 0
\end{aligned}$$

Let's assign $u_i \geq 0, i \in \{1, 2, 3, 4, E, SE, S, W, NW, N\}$ to each inequality constraint. Then the KKT conditions are

$$\begin{cases} -1 = 2u_1 + 2u_2 - u_3 - u_E \\ -1 = u_1 + u_2 - u_3 + u_4 - u_{SE} \\ -1 = -u_1 - u_2 + u_4 - u_S \\ -1 = -2u_1 - 2u_2 + u_3 - u_W \\ -1 = -u_1 - u_2 + u_3 - u_4 - u_{NW} \\ -1 = u_1 + u_2 - u_4 - u_N \\ \quad 0 \geq 2v_E + v_{SE} - v_S - 2v_W - v_{NW} + v_N + (2r_0 + r_1) - \lfloor x_L \rfloor \\ \quad 0 \geq 2v_E + v_{SE} - v_S - 2(v_W - 1) - v_{NW} + v_N + (2r_0 + r_1) - \lfloor x_L \rfloor - 1 \\ \quad 0 \geq -v_E - v_{SE} + v_W + v_{NW} - r_0 + r_0^{\min} \\ \quad 0 \geq v_{SE} + v_S - v_{NW} - v_N - r_1 + r_1^{\min} \\ \quad 0 = u_1\{2v_E + v_{SE} - v_S - 2v_W - v_{NW} + v_N + (2r_0 + r_1) - \lfloor x_L \rfloor\} \\ \quad 0 = u_2\{2v_E + v_{SE} - v_S - 2(v_W - 1) - v_{NW} + v_N + (2r_0 + r_1) - \lfloor x_L \rfloor - 1\} \\ \quad 0 = u_3 \left(-v_E - v_{SE} + v_W + v_{NW} - r_0 + r_0^{\min}\right) \\ \quad 0 = u_4 \left(v_{SE} + v_S - v_{NW} - v_N - r_1 + r_1^{\min}\right) \\ \quad 0 = u_E v_E = u_{SE} v_{SE} = u_S v_S = u_W v_W = u_{NW} v_{NW} = u_N v_N \\ \quad 0 \leq u_i, i \in \{1, 2, 4, E, SE, S, W, NW, N\} \end{cases}$$

Because the objective function is concave and the inequality constraints are convex, the KKT conditions are sufficient for optimality. The following points satisfy the KKT conditions, and hence they are solutions to the optimization problem:

(i) When $2\left(r_0 - r_0^{\min}\right) \geq (2r_0 + r_1) - \lfloor x_L \rfloor$ and $(2r_0 + r_1) - \lfloor x_L \rfloor$ is even:

$$\begin{aligned} (v_E, v_{SE}, v_S, v_W, v_{NW}, v_N) &= (0, 0, 0, \frac{(2r_0 + r_1) - \lfloor x_L \rfloor}{2}, 0, 0) \\ (u_1, u_2, u_3, u_4) &= (\frac{1}{2}, 0, 0, 0) \\ (u_E, u_{SE}, u_S, u_W, u_{NW}, u_N) &= (2, \frac{3}{2}, \frac{1}{2}, 0, \frac{1}{2}, \frac{3}{2}) \end{aligned}$$

(ii) When $2\left(r_0 - r_0^{\min}\right) \geq (2r_0 + r_1) - \lfloor x_L \rfloor$ and $(2r_0 + r_1) - \lfloor x_L \rfloor$ is odd:

$$\begin{aligned} (v_E, v_{SE}, v_S, v_W, v_{NW}, v_N) &= (0, 0, 0, \left\lceil \frac{(2r_0 + r_1) - \lfloor x_L \rfloor}{2} \right\rceil, 0, 0) \\ (u_1, u_2, u_3, u_4) &= (0, \frac{1}{2}, 0, 0) \\ (u_E, u_{SE}, u_S, u_W, u_{NW}, u_N) &= (2, \frac{3}{2}, \frac{1}{2}, 0, \frac{1}{2}, \frac{3}{2}) \end{aligned}$$

(iii) When $2\left(r_0 - r_0^{\min}\right) < (2r_0 + r_1) - \lfloor x_L \rfloor$:

$$\begin{aligned} v_W &= r_0 - r_0^{\min} \\ v_S &= (2r_0 + r_1) - \lfloor x_L \rfloor - 2v_W \\ (v_E, v_{SE}, v_{NW}, v_N) &= (0, 0, 0, 0) \\ (u_1, u_2, u_3, u_4) &= (1, 0, 1, 0) \\ (u_E, u_{SE}, u_S, u_W, u_{NW}, u_N) &= (2, 1, 0, 0, 1, 2) \end{aligned}$$

That is, the optimal solution can be found by either

(1) increasing v_W until a solution is found, if $2\left(r_0 - r_0^{\min}\right) > (2r_0 + r_1) - \lfloor x_L \rfloor$, or

(2) increasing v_W until $v_W = r_0$ then decreasing v_S until a solution is found, if $2\left(r_0 - r_0^{\min}\right) < (2r_0 + r_1) - \lfloor x_L \rfloor$.

Similarly, we can find the shortest Hamming distance from the table D, represented by the point (r_0, r_1), to the shaded area to the right of the black linesby solving the following optimization problem:

$$\begin{aligned} \text{minimize:} \quad & v_E + v_{SE} + v_S + v_W + v_{NW} + v_N \\ \text{subject to:} \quad & 2v_E + v_{SE} - v_S - 2v_W - v_{NW} + v_N \geq +\lceil x_R \rceil - (2r_0 + r_1) \\ & 2(v_E - 1) + v_{SE} - v_S - 2v_W - v_{NW} + v_N < \lceil x_R \rceil - (2r_0 + r_1) \\ & \frac{(r_1 + v_N + v_{NW} - v_S - v_{SE}) - r_1^{\max}}{(r_0 + v_E + v_{SE} - v_W - v_{NW}) - r_0^{\min}} \leq \frac{r_1^{\min} - r_1^{\max}}{r_0^{\max} - r_0^{\min}} \\ & v_E, v_{SE}, v_S, v_W, v_{NW}, v_N \geq 0, \end{aligned}$$

Where $r_i^{\max}$ and $r_i^{\min}$ are, respectively, the maximum and minimum values r_i can take. The first constraint ensures that D crosses or ends up on the black line on the right. The second constraint ensures that D does not end up too far from the black line; i.e., moving D to the west by 1 step will prevent D from crossing the black line. The third constraint ensures that D stays inside the grid and does not move past the line $\frac{y - r_1^{\max}}{x - r_0^{\min}} = \frac{r_1^{\min} - r_1^{\max}}{r_0^{\max} - r_0^{\min}}$, which in Figure 2 is the top-right boundary formed by connecting the right most dots for each r_1. Once again, the KKT conditions are sufficient for optimality. Let's assign $u_i \geq 0, i \in \{1, 2, 3, E, SE, S, W, NW, N\}$ to each inequality constraint, then the following points satisfy the KKT conditions:

(i) When $\frac{r_0^{\max} - r_0^{\min}}{r_1^{\max} - r_1^{\min}} \left(r_1^{\max} - r_1\right) - \left(r_0 - r_0^{\min}\right) \geq \frac{(2r_0 + r_1) - \lceil x_R \rceil}{2}$ and $(2r_0 + r_1) - \lceil x_R \rceil$ is even:

$$\begin{aligned} (v_E, v_{SE}, v_S, v_W, v_{NW}, v_N) &= \left(\frac{(2r_0 + r_1) - \lceil x_R \rceil}{2}, 0, 0, 0, 0, 0\right) \\ (u_1, u_2, u_3) &= \left(\frac{1}{2}, 0, 0\right) \\ (u_E, u_{SE}, u_S, u_W, u_{NW}, u_N) &= \left(0, \frac{1}{2}, \frac{3}{2}, 2, \frac{3}{2}, \frac{1}{2}\right) \end{aligned}$$

(ii) When $\frac{r_0^{\max}-r_0^{\min}}{r_1^{\max}-r_1^{\min}}\left(r_1^{\max}-r_1\right)-\left(r_0-r_0^{\min}\right) \geq \frac{(2r_0+r_1)-\lceil x_R \rceil}{2}$ and $(2r_0+r_1)-\lceil x_R \rceil$ is odd:

$$
\begin{aligned}
(v_E, v_{SE}, v_S, v_W, v_{NW}, v_N) &= (\left\lceil \frac{(2r_0+r_1)-\lceil x_R \rceil}{2} \right\rceil, 0, 0, 0, 0, 0) \\
(u_1, u_2, u_3) &= (0, \frac{1}{2}, 0) \\
(u_E, u_{SE}, u_S, u_W, u_{NW}, u_N) &= (0, \frac{1}{2}, \frac{3}{2}, 2, \frac{3}{2}, \frac{1}{2})
\end{aligned}
$$

(iii) When $\frac{r_0^{\max}-r_0^{\min}}{r_1^{\max}-r_1^{\min}}\left(r_1^{\max}-r_1\right)-\left(r_0-r_0^{\min}\right) < \frac{(2r_0+r_1)-\lceil x_R \rceil}{2}$:

$$
\begin{aligned}
v_E &= \frac{r_0^{\max}-r_0^{\min}}{r_1^{\max}-r_1^{\min}}\left(r_1^{\max}-r_1\right)-\left(r_0-r_0^{\min}\right) \\
v_{SE} &= (2r_0+r_1)-\lceil x_R \rceil - 2v_E \\
(v_S, v_W, v_{NW}, v_N) &= (0, 0, 0, 0) \\
u_1 &= \frac{1}{2} + \frac{\left(r_1^{\max}-r_1^{\min}\right)/2}{2\left(r_0^{\max}-r_0^{\min}\right)-\left(r_1^{\max}-r_1^{\min}\right)} \\
u_2 &= 0 \\
u_3 &= \frac{1}{2\left(r_0^{\max}-r_0^{\min}\right)-\left(r_1^{\max}-r_1^{\min}\right)} \\
(u_E, u_{SE}, u_S, u_W, u_{NW}, u_N) &= (0, 0, 1, 2, 2, 1)
\end{aligned}
$$

That is, the optimal solution can be found by either

(1) increasing v_E until a solution is found, if $\frac{r_0^{\max}-r_0^{\min}}{r_1^{\max}-r_1^{\min}}\left(r_1^{\max}-r_1\right)-\left(r_0-r_0^{\min}\right) \geq \frac{(2r_0+r_1)-\lceil x_R \rceil}{2}$, or

(2) increasing v_E until $v_E = \frac{r_0^{\max}-r_0^{\min}}{r_1^{\max}-r_1^{\min}}\left(r_1^{\max}-r_1\right)-\left(r_0-r_0^{\min}\right)$, then decreasing v_{SE} until a solution is found, if $\frac{r_0^{\max}-r_0^{\min}}{r_1^{\max}-r_1^{\min}}\left(r_1^{\max}-r_1\right)-\left(r_0-r_0^{\min}\right) < \frac{(2r_0+r_1)-\lceil x_R \rceil}{2}$.

□
